# Enriching health-professional programs in global health: Development and implementation of an interdisciplinary and integrated approach

**Published:** 2017-04-20

**Authors:** Carol Valois, Véronique Foley, Paul Grand’Maison, Johanne Dumont

**Affiliations:** 1 Faculty of Medicine and Health Sciences, Université de Sherbrooke, Quebec, Canada

## Abstract

**Background:**

Globalization results in a rapidly diversifying population, increased inequities, and more complex health problems affecting populations. This forces medical schools to integrate global health (GH) into the training of health-care professionals from curriculum development to practical learning activities, here and abroad.

**Approach:**

The approach aims at enriching existing programs in GH competencies in an interdisciplinary context. The goal is to ensure that all health-science students develop a certain level of GH competency. The main actions are the mobilization of key stakeholders, the development of a competency framework (CF) to perform gap analysis, tool formalization, and monitoring and evaluation activities. Subsequent to scoping review and stakeholder consultations, ten principles are identified and used to guide the enrichment process.

**Results:**

Actual outputs cover a broad scope, from key decision-makers’ support and endorsement to the formalization of tools and the consolidation and creation of activities such as service-learning activities, rotations among underserved populations, and training for international rotations.

**Conclusion:**

While this unique approach is proving to be a major challenge, the preliminary results are well worth the effort. The project’s tangible impacts on health-sciences teaching, the GH competence of graduates, and care delivery are topics of interest for future investigation.

## Background

Local, national, and international news provide us daily reminders that we live in a globalized world with increased interaction and integration of human, societal, and economic activities around the world and among countries.^1^ Globalization influences population mobility and human diversity, impacting health by spreading diseases and modifying disease burdens for populations and individuals.^2^,^3^ It clearly shows health inequities due to social determinants of health and the presence of numerous vulnerable populations,^4^ both of which are unfortunately growing in most countries. Such are the priority issues in global health (GH) as defined by Koplan et al.,^5^ and health-care professionals in all disciplines have to take them into account to provide adequate and socially accountable care to the population served.^6^

Educational institutions for health-care professionals are consequently called upon to integrate GH issues into their programs.^7^–^10^ They are urged to define their graduates’ expected GH exit competencies and ensure their development throughout curriculum change, modified educational practices, and research. The overall aim is to improve the health of all individuals and populations at home and abroad by addressing issues such as diversity and inequities and their impacts on health.^11^

Academic institutions are under external pressure to move in this direction. In addition, many students are showing growing interest in GH and want to be better equipped to care for the most vulnerable individuals,^12^,^13^ yet often feel ill prepared to do so.^14^

Health-sciences programs are often asked to add new curriculum items or content.^15^ Heavily loaded curricula, time and space limitations, and limited GH resources and expertise have led many institutions to offer GH training as an elective for interested students, often at the graduate level.^16^,^17^ Regardless of the quality of such training, it fails to respond to the goal of having all students develop GH core competencies. Furthermore, disciplines must collaborate to better address the complexity of the issues and to ensure higher quality of care in GH, calling for an interdisciplinary educational approach, which is a significant challenge in itself.

Over the last four years, the Université de Sherbrooke’s Faculty of Medicine and Health Sciences (FMHS) has committed itself to overcoming these challenges. Herein, we share FMHS’s vision and actions for an interdisciplinary and integrated approach to enriching health-sciences programs in GH. It particularly focuses on underlying principles, project implementation processes, and our preliminary results.

The foundation for this work lies with the FMHS strategic plan to develop social accountability and competencies in GH for future health professionals.^18^ The initial reflection on enriching undergraduate programs with GH concepts was initiated by a GH-expert faculty member who had already been involved in international rotations for many years and who was coordinating a six-credit graduate program in international health for medical residents, practicing physicians, and nurses in preparation for such rotations. The budding idea was quickly supported by the new leadership of the FMHS Office of International Relations with a commitment to develop GH education beyond simple pre-departure orientations. Several other isolated GH initiatives were in place within the FMHS; student interest was growing. Institutional commitment and individual efforts converged, and the time had come to implement the current comprehensive project.

## Approach

The *project goal* is to ensure that all health-care professionals are committed to GH, socially responsible, and more competent at serving vulnerable or excluded populations.

**Figure 1:**
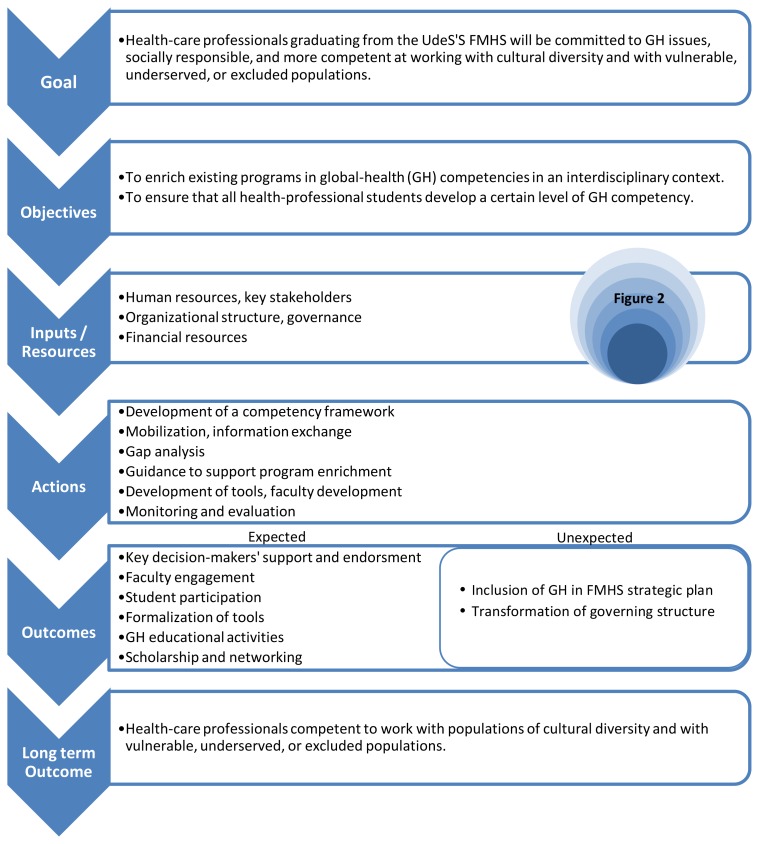
Logic model framework used to structure the project

The *main objectives* are to enrich the four existing programs (medicine, occupational therapy, physiotherapy, and nursing sciences) in GH competencies in an interdisciplinary context to ensure that all health-sciences students develop a level of GH competency adequate for addressing the challenges of practicing in a globalizing world. The logic model framework (LMF) illustrated in Figure 1 was used in the development and planning phases. The sections below were structured according to elements of this model.

The project consists of developing and implementing GH competency in existing programs. Given its nature, no ethical approval was required for this project.

A scoping literature review of GH issues in health and health-sciences education programs over the last ten years was conducted. Broad consultations were also performed to round out our thinking and further develop the characteristics of the educational approach and to ensure broader acceptance. Key practitioners in the field (in-house clinicians or teachers involved in GH practice, teaching, or research; members of North American and European organizations met at various conferences on social accountability), students interested in GH, leaders of health-professional education programs, and educational specialists were consulted during roundtable discussions. All were asked about students to be targeted; vision of GH education and training to be developed at our school; existing activities and resources; program coverage of issues such as diversity, social determinants of health, and vulnerable and underserved populations; and suggestions on how to integrate GH into programs; etc. Educational and organizational principles were progressively confirmed. Box 1 presents those that are constantly guiding our work.

Box 1 **Guiding principles for GH education resulting from consultations**

- The main focus is on enriching existing learning activities rather than adding to the curriculum, while supporting new initiatives, when needed.
- GH competencies are closely linked to and complement the exit competencies of each program.
- The educational interventions are simultaneously and conjointly implemented in the FHMS’s four health-professional programs: medicine, nursing, physiotherapy, and occupational therapy.
- All students in the targeted programs develop a basic level of GH competencies (mandatory basic profile) through access to core GH training.
- Advanced training activities are available for interested students to develop competencies at a higher level (advanced profile).
- GH education must reflect GH’S local and international issues.
- The exit purposes and competencies are common to all four programs but adapted to each discipline.
- The methods to ensure implementation are adapted to the specific realities and contexts of each program and are flexible enough to be customized as needed. They include both theoretical and practical activities in large or small groups, service-learning activities, clinical rotations, etc. They occur throughout the four-year programs.
- Collaboration between disciplines is fostered through interdisciplinary educational activities.
- Learning activities are of increasing complexity from the first to fourth year (stepladder approach).

### Inputs and resources

**Figure 2:**
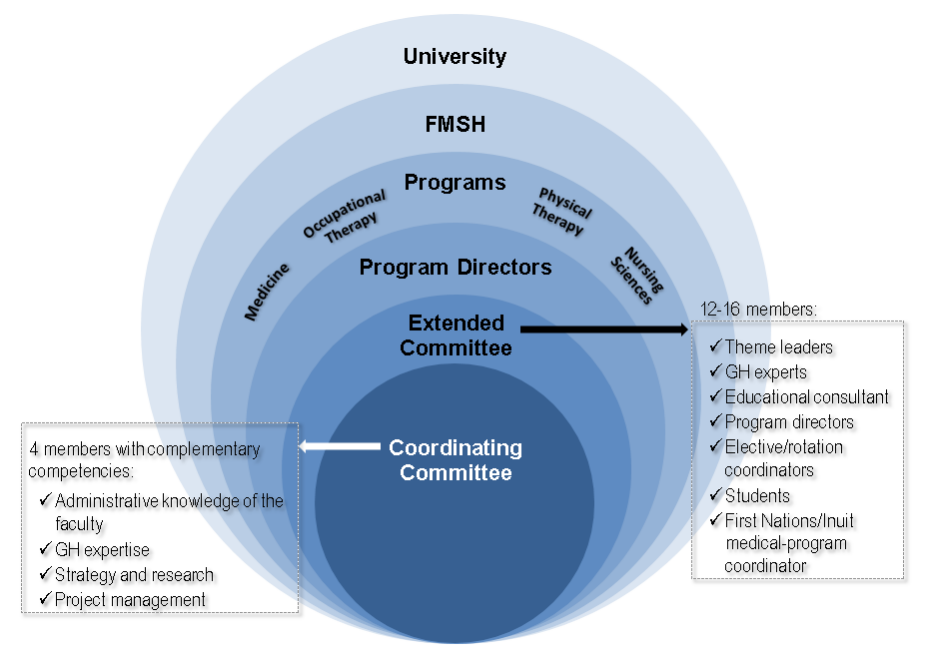
Organizational structure

Coordinating this project covering four (4) programs and more than 1,650 students represents a major organizational challenge, due, in part, to the number of stakeholders involved. Figure 2 presents the intertwined levels of the organizational structure.

A *coordinating committee*, ensuring overall project coordination, has four members (the authors of this paper) with complementary expertise and competencies: a professor with major GH expertise in practice and education, a senior professor with extensive administrative knowledge of the FMHS’s inner political workings, an occupational therapist with clinical and research experience, and a professional project coordinator. This committee meets for a full day on a bimonthly basis with the objectives of ensuring a comprehensive vision of GH training within the FMHS, supporting programs in implementing GH enrichment, advocating for GH education and the enrichment project, obtaining the required resources, and confirming and supporting partnerships. Program directors appointed one faculty member involved in their programs and committed to GH to act as **GH theme leaders** (TLs) for the program. Together with the coordinating committee, these individuals form the **extended committee** which also includes a student representative from each program, a medical-education expert, a program-evaluation expert, clinical-rotation coordinators, and the MD-program faculty member responsible for ensuring consideration is taken of Quebec’s First Nations and Inuit peoples. While opinions vary on including Indigenous people in GH, we feel it is important to consult someone knowledgeable about the historical and contextual aspects related to First Nations and not to focus solely on their health problems. This is invaluable in providing an appropriate context for clinical scenarios and avoiding stereotyping.

Program directors attend the committee’s meetings for consultation at strategic times or when their engagement as leaders is important in implementing project actions. The **extended committee** meets for three hours four times a year in a context of interdisciplinary collaboration. It acts as an intermediary between the coordinating committee, programs, and students, while providing the coordinating committee with suggestions on enhancing programs.

The Université de Sherbrooke, the FMSH, and the Office of International Relations provide engagement and support in the form of financial and human resources. A sustainability plan is in place to ensure continuous funding, support from FMHS stakeholders as well as commitment from program directors and education leaders.

### Actions

An internal communication plan targeting FMSH deans, program directors, teachers, and students helps bring all stakeholders on board and get them to buy in. The TLs are highly instrumental in creating crucial bridges with their respective programs. As project ambassadors, they provide influential leadership in adopting the project’s goal and objectives and adapting actions to their specific needs and characteristics. Adaptability and flexibility are proven key success factors.

The targeted programs use the GH competency framework—developed and generally accepted at Sherbrooke FMHS and adapted to CanMEDS roles^19^ —to identify the GH aspects (competencies, content, attitudes, and skills) explicitly or implicitly covered in the curricula and those to be developed. Concretely, this three-step process analyzes the GH aspects already covered, analyzes the gap between the actual and targeted coverage (as described in the GH competency framework), and integrates missing GH aspects. This gap-analysis approach encourages programs to integrate new GH elements, taking into account the existing content while striving to avoid curriculum overload.

Collaborative work and consultation with experts resulted in the development, adoption, and uptake of various tools within our faculty. These tools facilitate communication and ensure the consistency of the work with and within each program.

Since faculty development is essential, a plan was developed and adapted to teacher needs, which are twofold: increase teacher GH competency (most teachers) and increase teacher capacity to optimally sustain student GH competency development (all teachers).

Educational and program-evaluation specialists assist in developing evaluation and monitoring plans so that project processes and implementation can be monitored on an ongoing basis. Monitoring consists of regular reports, action documentation, and qualitative consultations with TLs, program directors, and students.

Box 2 **Tools developed as a result of the committees’ work**

- **An operational definition^a^ of Global Health** adapted to our learning environment.
- **A competency framework** that describes in detail the key elements and interdisciplinary learning targets (targeted competencies) using commonly agreed-upon terminology. Inspiration for this framework came from CanMEDS roles,^b^ AFMC recommendations,^19^ and a literature review. It has been adapted to the Professional Development Path model used at the Université de Sherbrooke for programs other than medicine.
- **A comprehensive vision of the FMHS GH training continuum** covering undergraduate medical education and other undergraduate programs, residency programs, graduate programs, continuing education, and faculty development.
- **A dedicated and continually updated collaborative intranet site** accessible to the whole FMHS community. It serves as the central depository of all documents and communications regarding project developments and includes a reference library of articles, related sites, and available GH resources.
- **A faculty-development program** designed to reach all concerned teachers and build collective GH competency within the FMHS.

aFMHS GH operational definition: *A field of study, research, and practice that transcends boundaries aimed at improving health and equity in health care for every individual worldwide. This takes into account the global burden of health problems; social and economic determinants; complementarily of the individual and population approaches; impacts related to the mobility of populations and individuals; problems associated with the health and well-being of travelers (tourists, workers, or individuals in an intervention context, e.g., military or humanitarian) and migrants; vulnerable populations; communities with limited resources; environmental concerns and the impact of climate change on health; concepts of marginality, exclusion, poverty, and vulnerability; and political, economic, social, national, and international issues that affect health*.bCanMEDS: *framework for improving patient care by enhancing physician training. Developed by the Royal College in the 1990s, its main purpose is to define the necessary competencies for all areas of medical practice and provide a comprehensive foundation for medical education and practice in Canada*.

## Results/outcomes

Through regular project updates, the FMHS dean, academic vice-deans, and program directors support the project and endorse its guiding principles. Participation in meetings and implementation of GH-enriched educational activities demonstrate that teachers are engaged and motivated to modify their teaching and educational activities. Adopting a student-centered approach — getting students involved in the process early on and regularly consulting them — keeps us aware of their needs. Four student members on the extended committee provide learner perspectives.

Below is a brief list of the tools developed as a result of the committees’ work.

Based on the gap analysis between the actual and targeted coverage of GH aspects (as described in the GH competency framework), programs review their learning activities to close the gap and meet the objectives set. Here are few examples of newly developed or consolidated initiatives:

- Seizing the opportunity: major curriculum renewal of the MD program. Various ways of incorporating GH concepts into new educational activities are progressively implemented.
- Occupational-therapy program: development and implementation of “*Société, culture et occupation,*”^20^ a course that deals with inequity and cultural issues.
- Implementation of service-learning activities targeting vulnerable populations in the community; required participation for all first- and second-year medical students. The competency framework is used to develop these activities in order to enhance cultural humility and community engagement.
- Clinical rotations for occupational-therapy students in daycares and community schools in low-resource settings.
- Optimization of pre-departure training available to all students going on international rotations to include GH key elements developed through this project. Now offered to students on rotations in GH contexts in local communities.
- Development of comprehensive revision of all issues (disciplinary, global health, security, etc.) to support students before, during, and after international and local GH rotations, based on Besette 2016 scoping review.^21^

Throughout the years, project leaders have implemented the communication plan by maximizing internal and external national/international dissemination activities. These actions ensure continuous clarification and exchange of ideas with other experts, on-going development of team expertise, partnerships with other medical schools and experts in the field, and scholarship.

The project has revealed **unexpected results**, such as the gradual transformation of health-professional education programs from a silo approach to a culture of collaboration. The work in developing the common competency framework is bringing together the various disciplines to share their professional perspectives. The **extended-committee** meetings offer unique occasions for program directors and faculty members to work together on a common project and openly share several initiatives in their respective programs.

The project convinced FMHS leadership to deepen its engagement for GH. In its *2016–2018 Strategic Plan*, the FMHS commits to enrich its programs so as to model, promote, and seek social accountability and to ensure GH-skill training for all future health-care professionals.

**Figure 3:**
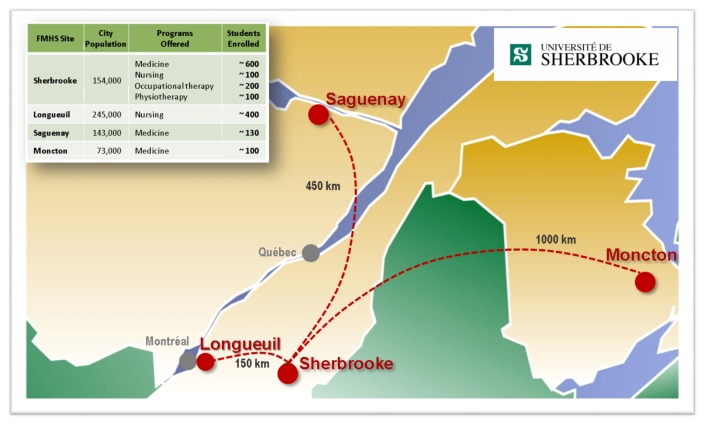
Campuses and site of the Faculty of Medicine and Health Sciences

The pace of enrichment varies according to the program and campus location (see Figure 3). Specific realities and contexts need to be taken into account. For example, the fully distributed medical-education programs in Moncton and Saguenay are closer to First Nations communities, which facilitates clinical rotations serving these populations.

## Discussion

A project of this magnitude cannot be implemented without challenges. Efficient governance through constant communication with all stakeholders is necessary to support programs through the implementation process, alleviate the resistance related to curriculum overload, and assist in identifying existing GH activities often not explicitly described in the curriculum.

The relationship of trust among stakeholders and the team buying into common goals mitigated some of these challenges by proactively resolving issues. Our organizational structure is based on strong leadership, intertwined responsibilities, connectivity among different levels, and efficient collaboration.^22^

Over the next three years, we intend to continue to support the gradual implementation of key GH elements within programs, clearly establish the optional advanced GH profile and provide means for student recognition, deploy faculty-development activities, and establish the GH connection from undergraduate programs to residency programs, master’s programs, and doctoral programs as well as in continuing-education activities. One of the most important aspects will be studying the project’s impact on student learning and student development of GH competencies.

### Conclusion

The project of enriching existing health-professional educational programs in GH competencies within an interdisciplinary context is a major undertaking. Nevertheless, the preliminary results are well worth the effort invested.

It might have been simpler to target a single program without considering an overall vision or consistency. Targeting the four programs yielded healthy discussions and unexpected benefits. Sharing led to mobilization and eventually to emulation. Involving stakeholders early on in developing the vision has proven effective in bringing people on board. Working closely with various programs is more demanding than simply bringing together GH experts, but it significantly increases buy-in and consequently speeds up implementation.

Taking our time, consulting stakeholders, and respecting paces is both wise and advantageous. Developing a common competency framework while allowing room for program readiness leads to its quick adoption. Sharing a common language and working collaboratively has positive impacts.

Having a determined, well-supported team convinced of the project’s importance is fundamental because the time and resources required to implement a project of this scale must not be underestimated.

So far, this project meets our ambitious original objectives. It establishes a vision and sets a framework that prepares students to become competent health-care professionals equipped to meet the challenges brought about by globalization. A qualitative study is currently being planned to determine the right paradigm to truly evaluate the impact on student learning and GH competencies. The project’s tangible impacts on health-sciences teaching, the GH competencies of graduates, and care delivery are topics of interest for future investigation.
